# Application of gene expression programming and neural networks to predict adverse events of radical hysterectomy in cervical cancer patients

**DOI:** 10.1007/s11517-013-1108-8

**Published:** 2013-10-18

**Authors:** Maciej Kusy, Bogdan Obrzut, Jacek Kluska

**Affiliations:** 1 Faculty of Electrical and Computer Engineering, Rzeszow University of Technology, W. Pola 2, 35-959 Rzeszow, Poland; 2 Faculty of Medicine, University of Rzeszow, Warszawska 26a, 35-205 Rzeszow, Poland

**Keywords:** Cervical cancer, Radical hysterectomy, Perioperative complications, Gene expression, Programming, Neural networks

## Abstract

The aim of this article was to compare gene expression programming (GEP) method with three types of neural networks in the prediction of adverse events of radical hysterectomy in cervical cancer patients. One-hundred and seven patients treated by radical hysterectomy were analyzed. Each record representing a single patient consisted of 10 parameters. The occurrence and lack of perioperative complications imposed a two-class classification problem. In the simulations, GEP algorithm was compared to a multilayer perceptron (MLP), a radial basis function network neural, and a probabilistic neural network. The generalization ability of the models was assessed on the basis of their accuracy, the sensitivity, the specificity, and the area under the receiver operating characteristic curve (AUROC). The GEP classifier provided best results in the prediction of the adverse events with the accuracy of 71.96 %. Comparable but slightly worse outcomes were obtained using MLP, i.e., 71.87 %. For each of measured indices: accuracy, sensitivity, specificity, and the AUROC, the standard deviation was the smallest for the models generated by GEP classifier.

## Introduction

Cervical cancer is the third most common malignant neoplasm of female reproductive organs. The estimated incidence is approximately 530,000 new cases yearly [27]. Operative methods, irradiation, and combined treatment consisting of surgical techniques, radiotherapy, and most recently chemotherapy are applied in the management of cervical cancers. The treatment choice is dependent first of all on the disease advancement. Advanced stages of cervical cancer are treated with radio-chemotherapy, but yet in the early developmental stages of cervical cancer, primary surgical treatment is preferred. In FIGO stages 0–IA1, the surgical treatment has limited spectrum (conization, cervical amputation, simple hysterectomy, or radical trachelectomy) [50]. Radical hysterectomy (i.e., removal of uterus along with suspensory ligamentous apparatus and vaginal cuff) with pelvic lymphadenectomy is the treatment of choice for cervical cancer in FIGO stages IA2–IIA (and some FIGO IIB cases) [25, 28]. This operative method, as a very extensive surgical procedure, is burdened with significant risk of complications ranging from 8 % up to 88 %, according to data from the literature [5, 26, 34, 47, 61]. In considerable part, these are non-onerous defecation dysfunctions, urinary tract infections, or transient urinary bladder atony [31, 34, 61]. However, in up to 6.6 % of cases, damage of urinary bladder comes about [31], and in 2.6 % of operated patients, iatrogenic damages of ureters occur [49]. Pulmonary artery embolism is a life-threatening complication, which occurs with approximately 1–1.5 % incidence [31, 49]. Adverse events of radical hysterectomy performed in cervical cancer are also pregnant with effects from other considerations. They constitute an additional burden to female patient, who is already in psychologically and often physically ill condition due to a neoplastic disease. Intraoperative complications often extort ending the surgical procedure before reaching sufficient range of tissue excision. Lack of oncological radicality in turn is the reason for implementation of adjuvant radiotherapy, which could be avoided at least in some patients successfully treated by surgery. Thus, adjuvant radiotherapy is often delayed till the time of complete recovery from perioperative complications, which is not without the influence on patients’ survival time. Moreover, complications following primary surgical treatment with subsequent radiotherapy are greater than those following primary radical radiotherapy [4] to which the patient could be initially scheduled if potentially threatening complications had been foreseeable. Factors influencing the occurrence of adverse events in gynecologic oncology were described well enough. Algorithm assessing the risk of complications pertaining to operative management in these patients was also elaborated [32]. Yet, in females with cervical cancer, the algorithm has limited application, because it does not take into account the neoplasm staging, which has the fundamental influence on the degree of difficulty of planned surgery. The aim of the study was to create the prediction model which, by the use the AI methods, allows to anticipate the occurrence of complications of radical hysterectomy in patients with FIGO IA2–IIB cervical cancer.

## Methods

### Study group

The prospective cohort study included 107 patients with cervical carcinoma, who were treated surgically at the State Hospital in Rzeszow during 1998–2001. The patients’ age range was 29–73 (median age was 48.60, with standard deviation *σ* = 9.88). A majority of them (71 patients) were in the reproductive period. The postmenopausal status was found in 36 patients. The mean value for the body mass index (BMI) in the study group was 26.09 kg/m^2^ (*σ* = 4.99). The clinical progression of cancer was assessed according to the FIGO criteria. The distribution of the cervical carcinoma stages in the study group is presented in Table 1. Histopathological diagnosis was based on directed cervical biopsy and fractionated abrasion. In disputable cases (17 patients), cervical conization was performed. The prevailing type was squamous cell carcinoma (89.72 %). Other histological forms were found in 11 patients (10.28 %). Concomitant diseases were found in 36 women (Table 1), while more than one accompanying disease occurred simultaneously in 5 patients. Some of the subjects (27 women) had received surgical treatment within the abdominal cavity in the past. Adverse events (perioperative complications) were assessed prospectively during the operation (intraoperative complications) and within 30 days following the surgery (postoperative complications).

**Table 1:** Preoperative data in the study group (*n* = 107)

Number of patients		107
Age (mean/*σ*)		48.60/9.88
Hormonal status		
Premenopausal		71
Postmenopausal		36
Body mass index (mean/*σ*)		26.09/4.99
Concomitant diseases		
Hypertension		26
Diabetes mellitus		3
Ischemic heart disease		9
Other		3
Previous abdominal surgeries		27
FIGO stage		
IA2		17 (15.89 %)
IB1		52 (48.60 %)
IB2		8 (7.48 %)
IIA		8 (7.48 %)
IIB		22 (20.56 %)
Histological type		
Squamous		96 (89.72 %)
Non-squamous		11 (10.28 %)
Grading		
G1		23 (21.50 %)
G2		64 (59.81 %)
G3		20 (18.69 %)

### Artificial intelligence methods applied

In the simulations, gene expression programming (GEP) algorithm was compared to three feedforward neural networks: the multilayer perceptron (MLP), the radial basis function neural network (RBFNN), and the probabilistic neural network (PNN). GEP algorithm and both radial basis function-based neural networks were simulated by DTREG software [51], while the MLP was trained using Statistica Data Miner [53].

#### Gene expression programming

GEP algorithm is an algorithm which, emulating biological evolution, creates and evolves computer programs. GEP was introduced by Ferreira [17] with the assumption of being, in some way, an extension of genetic programming (GP) [33] preserving few properties of genetic algorithms (GA) [21]. In contrast to GP, the chromosomes in GEP are not represented as trees, but as linear strings of fixed length, this, in turn, is the feature taken from GA. In GEP, the programs (individuals) are encoded by the chromosomes, which are composed of the genes structurally organized in the head and the tail. The length of genes is an open choice and depends on the head size. When the representation of each gene is given, the genotype is established. It is then converted to the phenotype—the expression tree (ET). In order to construct the chromosome, the genes are linked with each other by means of the linking function. Assumed number of these individuals forms the sample population which undergoes evolution by computing the expression from each chromosome, applying predefined genetic operators and calculating the fitness. The type of the fitness function is dependent upon the considered problem. Diverse genetic operators are used both within and between the chromosomes. The evolution continues until a termination criterion is satisfied [18].

For the cervical cancer complication prediction model, the GEP’s settings are shown in Table 2. In all simulations, the number of chromosomes in population was set to 30. For genetic computations, we used 10 random floating point constants per gene, from the range [−1,000, 1,000]. Evolution was performed until 1,000 generations were reached.

**Table 2:** The head size, the number of genes within each chromosome, the linking functions between genes, the computing functions in the head, the fitness functions and the genetic operators utilized for GEP model

Head size	2, 3, 4, 5, 6, 7, 8
Number of genes	$1,2,\ldots,15$
Linking function	Addition, multiplication, logical OR
Computing functions	+, −, $\ast$, /, −*x*, 1/*x*
	$$\sin(ax-b), \cos(ax-b)$$
	$$b/(1+\exp(ax)),\, \exp\left(-(x-a)^{2}/(2b^{2})\right)$$
Fitness function	Sensitivity/specificity
	Number of hits with precision
	Number of hits with penalty
	Mean squared error
Genetic operators	Mutation = 0.044
	Inversion = 0.1
	IS transposition = 0.1
	RIS transposition = 0.1
	Gene transposition = 0.1
	One-point recombination = 0.3
	Two-point recombination = 0.3
	Gene recombination = 0.1

#### Multilayer perceptron

MLP is the type of a neural network where the input signal is fed forward through a number of layers [48]. One can distinguish three types of layers in MLP: an input layer, at least one hidden layer, and an output layer. The input layer is composed of the elements, which are the features of an input pattern. The hidden layer consists of a predefined number of nodes called neurons. A particular hidden neuron adds all the values of input data variables multiplied by the weights and uses this weighted sum as its input. Such a signal is used as the argument of a transfer function of a hidden neuron. The output of each hidden neuron is distributed to all elements in the next layer. The output layer is composed of the neurons, which determine the final response of the model. This response is computed in the same way as the neuron’s output in the preceding layer. In the analysis, MLP composed of one or two hidden layers was used. The hidden and output layers were activated by the transfer functions from the set: {linear, hyperbolic tangent, logistic, exponential}. The number of hidden layer neurons was optimized in order to minimize the network error. Three MLP training algorithms were used: Broyden–Fletcher–Goldfarb–Shanno [6], a scaled conjugate gradient [41], and a traditional gradient descent algorithm.

#### Radial basis function neural network

RBFNN is a model in which the input signal is transmitted forward to the output node [10]. RBFNN consists of three layers: an input layer, a radial basis hidden layer and a linear output layer. In the input layer, there is one neuron for each predictor variable. The hidden layer is composed of *n* neurons of a radial basis functions centered on an input vector. The *n* number of neurons is determined during the training process. In this work, an evolutionary approach proposed by Chen et al. [12] was used to find an optimal *n*. The signal computed by the hidden layer is transmitted forward to the next linear layer. The linear layer calculates the weighted sum of the hidden layer outputs. For the classification problems, there are two nodes in the output layer, which represent a target category. The second layer weights are determined using ridge regression.

#### Probabilistic neural network

PNN is a feedforward model proposed by Specht [52], which is a direct implementation of Bayes classifier. In contrast to MLP and RBFNN, PNN is composed of four layers: an input layer, a pattern layer, a summation layer, and an output layer. In the input layer, there is one neuron for each data attribute. The pattern layer consists of the number of neurons equal to the cardinality of the training data set. Each neuron in this layer computes the Euclidean distance between the training pattern and the test case, and the resulting value undergoes the activation by the radial basis function. The signals coming from the pattern nodes, which belong to the class *c*, are summed and create single *c*th neuron in the summation layer. Thus, there are *C* neurons in the summation layer, where *C* denotes the number of classes. In the output layer, a decision is made on a final target for a test case. It is based on the largest value between the signals determined among all summation neurons. In the simulations, single smoothing parameter for each predictor variable is used. The parameter is computed using the conjugate gradient method [24].

## Results

The prediction ability of tested models was determined by computing the accuracy (Acc), the sensitivity (Sen), the specificity (Spe), and the area under the receiver operating characteristic curve (AUROC) [23] for the compared models: GEP, MLP, RBFNN, and PNN. All the performance indices were measured on the independent data subsets randomly extracted from the entire database, which comprise the following: 10, 20, and 30 % of the total number of patterns. It is worth to note that all the indices were computed for different parameters of the particular models.

Perioperative complications occurred in 47 patients: intraoperatively in 4 cases and postoperatively in remained 43 cases. Majority of these were mild or medium degree complications that did not pose a threat to the patient’s health or life. Severe perioperative complications (bleeding from the inferior vena cava, pulmonary embolism, gastric ulcer rupture, genitourinary fistulae) were found in 7 patients (Table 3). In the simulations, a binary classification was considered, i.e., occurrence or lack of complications.

**Table 3:** Complications in the study group (*n* = 107)

Complications	Number of patients	Incidence (%)
Intraoperative complications		
Urinary tract injury	2	1.87
Vena cava inferior injury	2	1.87
Total	4	3.74
Postoperative complications		
Acute cardiopulmonary symptoms	2	1.87
Femoral nerve injury	1	0.93
Abdominal wound infection or hematoma	5	4.67
Genitourinary fistula	3	2.80
Duodenal ulceration requiring surgery	1	0.93
Acute digestive symptoms	2	1.87
Asymptomatic lymphocele	3	2.80
Fever	10	9.35
Pulmonary embolism	1	0.93
Urinary retention	15	14.02
Total	43	40.19

On the basis of considered input data, it was possible to find the models, which predict the occurrence of perioperative complications of radical hysterectomy in patients with cervical cancer.

In Tables 4, 5, 6, and 7, the values of the performance indices: Acc, Sen, Spe, and AUROC are presented for all investigated classifiers. Due to the fact that these indices were computed for different training and test subsets, their values need to be averaged (we use $\overline{(\cdot)}$ symbol for arithmetic means). Furthermore, for the particular classifiers, it is necessary to calculate the standard deviations *σ*_(·)_. The last rows in all tables provide the “minimal indices” values which make the classifier acceptable as the predictive model. Below, we present the conclusions.

**Table 4:** Accuracy computed for GEP, MLP, PNN, and RBFNN

Test size (%)	Acc (%)			
	GEP	MLP	PNN	RBFNN
10	80.00	90.00	63.64	54.55
20	76.19	80.95	61.91	66.67
30	71.88	71.87	62.50	65.63
$\overline{{\hbox{Acc}}}$	76.02	80.94	62.68	62.28
$\sigma_{\rm Acc}$	4.06	9.07	0.88	6.72
$\overline{{\hbox{Acc}}}-\sigma_{\rm Acc}$	71.96	71.87	61.80	55.57

### Accuracy

**Fig. 1:**
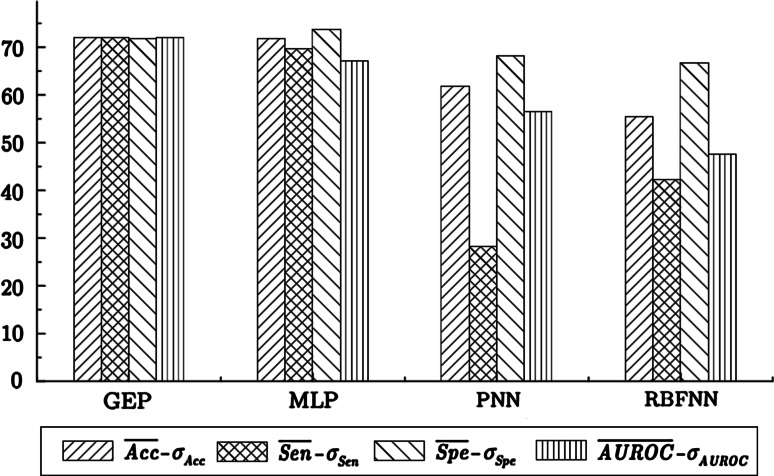
The “minimal values” of Acc, Sen, Spe, and AUROC in the prediction of adverse events in patients with cervical cancer

As shown in Table 4, the highest accuracy out of all compared models was found for the MLP classifier: $\overline{{\hbox{Acc}}}=80.94\,\%$. However, the standard deviation for this classification method is high: *σ*_Acc_ = 9.07 %, therefore $(\overline{{\hbox{Acc}}}-\sigma_{{\rm Acc}})_{{\rm MLP}}=71.87\,\%$. Hence, the “minimal accuracy” of MLP models is smaller than the one obtained for GEP classifiers: $(\overline{{\hbox{Acc}}}-\sigma_{\rm Acc})_{\rm GEP}=71.96\,\%$, despite the fact, that the average accuracy in case of GEP equals 76.02 %. That is because the standard deviation for GEP models is low: *σ*_Acc_ = 4.06 %. Thus, considering the accuracy measure, GEP and MLP methods generate similar models, which are much better than the remaining neural networks. The minimal values of accuracy determined for GEP, MLP, PNN, and RBFNN are illustrated in the form of the bar charts in Fig. 1.

### Sensitivity, specificity, and area under receiver operating characteristics

On the basis of Table 5, we infer that in case of sensitivity index, MLP procedure generates networks with a very high standard deviation: *σ*_Sen_ = 15.40 %, which is the largest among all tested models. Therefore, the “minimal sensitivity” for these networks equals $(\overline {{\hbox{Sen}}}-\sigma_{{\rm Sen}})_{\rm MLP}=69.58\,\%.$ For GEP models, we obtain a better outcome since the “minimal sensitivity” for these classifiers equals 71.95 %, which is a result of low standard deviation *σ*_Sen_ = 4.45 %. The sensitivity values of both radial basis function-based neural networks (PNN and RBFNN) are very low what utterly disqualifies these models.

**Table 5:** Sensitivity computed for GEP, MLP, PNN, and RBFNN

Test size (%)	Sen (%)			
	GEP	MLP	PNN	RBFNN
10	80.00	100.00	60.00	40.00
20	77.78	85.71	33.33	66.67
30	71.43	69.23	35.71	64.28
$\overline{{\hbox{Sen}}}$	76.40	84.98	43.01	56.98
*σ*_Sen_	4.45	15.40	14.76	14.76
$\overline{{\hbox{Sen}}}-\sigma_{\rm Sen}$	71.95	69.58	28.25	42.23

As presented in Table 6, the “minimal specificity” of all models reaches similar values. For MLP networks, both average and “minimal specificity” are the highest.

**Table 6:** Specificity computed for GEP, MLP, PNN, and RBFNN

Test size (%)	Spe (%)			
	GEP	MLP	PNN	RBFNN
10	80.00	83.33	66.67	66.67
20	75.00	78.57	83.33	66.67
30	72.22	73.68	83.33	66.67
$\overline{{\hbox{Spe}}}$	75.74	78.53	77.78	66.67
*σ*_Spe_	3.94	4.83	9.62	0.00
$\overline{{\hbox{Spe}}}-\sigma_{\rm Spe}$	71.80	73.70	68.16	66.67

The results in Table 7 show that both the average and the “minimal value” under the receiver operating characteristic are the highest for GEP models.

It is also worth to note that for each of measured indices: Acc, Sen, Spe, and AUROC, the standard deviation is smaller for the models generated by GEP classifier in comparison with MLP networks.

The results of “minimal values” of Sen, Spe, and AUROC for all tested models are summarized in Fig. 1. On the basis of the above analysis, we infer that the GEP classifier provides the best results in the prediction of the adverse events in cervical cancer patients treated by radical hysterectomy. Slightly worse outcomes are obtained using MLP neural network [30].

### Mathematical expression generated by GEP classifier

The results achieved by GEP, which are presented in Tables 4, 5, 6, and 7, are not the only outcome obtained by this algorithm. This evolutionary computation method generates a mathematical expression (a function), which fits the data with the accuracy obtained after the evolution process. Since in our investigation the “minimal prediction accuracy” of GEP equals 71.96 %, we provide the formula of such an expression found for Acc = 71.88 % where the training and test set sizes are equal 70 and 30 %, respectively. The mathematical function following from the Karva language expression [18] that solves the prediction problem takes the form:

1 $$\begin{aligned} f(x_{1},x_{2},x_{3},x_{4},x_{5}) &=\frac{7.98}{1+\exp(7.98\times x_{1}) }\times\frac{1}{1+\exp(-15.95\times x_{1})}\\ &\quad +x_{2}-11.74+\frac{1}{2}\times\frac{x_{3}}{1+\exp(x_{3}\times x_{4})}-x_{5}, \end{aligned}$$

where *x*_1_ is a binary representation of the BMI such that:

$$x_{1}=\left\{ \begin{array}{ll} 1, &\quad \hbox{if BMI is from the class ``underweight''}\\ 0, &\quad \hbox{otherwise} \end{array} \right.,$$

*x*_2_ is a binary representation of the FIGO stage and:

$$x_{2}=\left\{\begin{array}{ll} 1, &\quad \hbox{if FIGO stage is from the class IB2}\\ 0, &\quad \hbox {otherwise} \end{array} \right.,$$

*x*_3_ is an integer number from the set $\{29,\ldots,73\}$ which denotes an age of a patient, and *x*_4_ is a binary representation of the BMI such that:

$$x_{4}=\left\{ \begin{array}{ll} 1, & \hbox{if BMI is from the class ``obesity II''}\\ 0, & \hbox {otherwise} \end{array} \right.,$$

*x*_5_ is a binary representation of the FIGO stage and:

$$x_{5}=\left\{\begin{array}{ll} 1, & \hbox{if FIGO stage is from the class IB1}\\ 0, & \hbox {otherwise} \end{array} \right. .$$

The function presented in (1) depends on five input variables and provides the expression for the occurrence of radical hysterectomy complications in patients with cervical cancer with the prediction accuracy of 71.88 %. If the value of $f(\cdot)>0$, then the occurrence of complications takes place, and there is no adverse events when $f(\cdot)\leqslant0$. The method of complication occurrence verification is straightforward. Suppose, in our test set, there are two records representing the input measured features of two patients (case 1 and case 2, respectively) shown in Table 8.

**Table 7:** The area under receiver operating characteristic curve computed for GEP, MLP, PNN, and RBFNN

Test size (%)	AUROC			
	GEP	MLP	PNN	RBFNN
10	0.82	0.78	0.57	0.47
20	0.76	0.74	0.61	0.62
30	0.72	0.67	0.66	0.58
$\overline{{\hbox{AUROC}}}$	0.77	0.73	0.61	0.56
*σ*_AUROC_	0.05	0.06	0.05	0.08
$\overline{{\hbox{AUROC}}}-\sigma_{{\rm AUROC}}$	0.72	0.67	0.56	0.48

**Table 8:** Two real medical cases with all input variables and an output class

Input variable	Case 1	Case 2
Age	33	62
Height (cm)	164	164
Weight (kg)	63	60
Body mass index (type)	Normal	Normal
Concomitant diseases	0	0
Previous abdominal surgeries	No	Yes
Hormonal status	Premenopausal	Postmenopausal
Histological type	Squamous	Squamous
FIGO stage	IA2	IIB
Grading	2	3
Complications	No	Yes

Then, for cases 1 and 2, we obtain the following results:

$$\begin{aligned} f_{{\rm case}\_1}\,=\,&f(0,0,33,0,0)=-1.495, \\ f_{{\rm case}\_2}\,=\,&f(0,0,62,0,0)=5.755. \end{aligned}$$

As shown, $f_{{\rm case}\_1}<0$ and $f_{{\rm case}\_2}>0$, therefore, for the case 1, GEP model predicts lack of complications, while for the case 2, the adverse events will occur. In both cases, this prediction is assessed with the accuracy 71.88 %. It is worth to notice that the above-predicted results correspond to the real output values. This simple example confirms our belief that we obtain the partially interpretable model. From the mathematical point of view, this model is unique and readable. However, GEP method does not provide the set of simple “if-then” rules, which could be read by a specialist using a medical language. Thus, the received model can be regarded as a gray box.

## Discussion

Despite the achievements of theoretical sciences and rapid technological progress, undesirable occurrences still accompany modern medical procedures. According to the latest analyses, the frequency rate for complications in patients treated for gynecologic neoplasms falls in the range 26–54 % [19, 32]. As more and more attention is paid to the issue of patients’ life quality [11], the prevention of undesirable occurrences becomes one of the priorities of proceedings [16].

It was believed for a long time that in order to avoid complications it was sufficient to eliminate potential risk factors. The known risk factors for morbidity and mortality related to surgical treatment include inter alia, patient’s old age, duration and type of surgical procedure, occurrence of accompanying diseases, or obesity [32]. Unfortunately, these factors are not subject to modification (e.g., age, concomitant chronic diseases), or as in the case of considerable loss of body weight, they require longer time. There is no doubt that the postponement of oncological procedures until the proper BMI value is reached may have an adverse effect on the prognosis.

Therefore, the only effective way seems to involve the reliable identification of the risk factors and choosing such a therapeutic option that would minimize the risk of undesirable occurrences. It is important since, according to the literature data, a considerable part of iatrogenic complications can be prevented [3, 57]. Such hypothesis has been confirmed in surgery, where within 10 years of the introduction of the risk assessment system, the percentage of complications was reduced by 27–45 % [29]. An attempt was made to establish a similar risk model taking into account patients with ovarian carcinoma [1, 2], but it was not widely approved [32]. It also turned out that the model deriving from general surgery cannot be effectively applied in women with genital neoplasms [15]. As a response to the above situation, Kondalsamy–Chennakesavan et al. [32] developed a risk assessment system in gynecologic oncology. This model makes it possible to estimate the probability rate for undesirable occurrences in the general population of patients with genital neoplasms; yet, it does not allow for distinguishing various types of risk related to complications in particular types of cancer with more accuracy. This system does not account for the progression of neoplastic disease either, which may considerably increase the difficulty of a procedure and have an impact on the risk of complications. The tumor stage, as one of the input parameters of the perioperative prediction model, was firstly considered by our research team.

Neural networks are more and more widely used in medical sciences [37, 42, 45, 54, 60]. In cardiology, they are used, inter alia, to assess the status of cardiovascular system [43], to predict the risk of coronary heart disease [35] in ECG analysis [36, 56] or echocardiography [59]. In neurology, neural networks are used to predict a response to pharmacological treatment in Alzheimer’s disease [39]. In radiology, neural networks are effectively used to support the diagnosis of breast tumors [58], lung tumors [22], or liver tumors [38]. Automatic cytological screening of cervical carcinoma is a flagship example of the application of neural networks [8]. Neural networks were also used to predict complications following some medical procedures, e.g., percutaneous endoscopic gastrostomy [55], gastrectomy in patients with gastric carcinoma [14], laparoscopic cholecystectomy [20], or the mortality rate after cardiosurgical procedures [44]. The results of studies using the artificial intelligence methods in biomedical sciences are varied. Much better results are obtained in research using objective measurement data, e.g., the parameters of mammographic image [58], CT image [13], or results of laboratory tests [40]. In situations with the participation of the so-called human factor, the obtained results are slightly worse. The sensitivity and specificity of the ANN model in predicting conversion to laparotomy in patients who received laparoscopic cholecystectomy were 67 and 99 %, respectively [20]. The accuracy of ANN in predicting postoperative complications in patients receiving operative treatment because of gastric carcinoma was 84.16 % [14]. In the study investigating the possibilities of predicting pathologic pressure drop in patients under general anesthesia, the sensitivity and specificity of the ANN model were 74.4 and 85.6 %, respectively, with the accuracy of 82.3 % [2].

In general, our results do not differ from above-cited works. The more detailed comparative analysis is impossible to perform, because similar reports regarding the prediction of perioperative complications of cervical cancer treatment have not been published yet.

Evolutionary computation methods have also been applied in medical domains. Pena-Reyes and Sipper [46] provide an overview of evolutionary algorithms such as GAs, GP or evolution strategies in medical diagnosis, prognosis, imaging, signal processing, planning, and scheduling. Artificial neural networks were confronted to GP algorithm in medical data mining problem by Brameier and Banzhaf [9], who compared the models in the classification of six repository data sets. On the basis of a GP system, an evolutionary predictive model was built, which can be applied to diagnose a chest pain [7]. However, to the best knowledge of the authors, the applications of gene expression programming in medicine domain have not been proposed yet.

The weakness of this study is a small number of data examples. However, it is necessary to emphasize that the collection of a significantly greater material is difficult nowadays, because of a decline in the overall incidence of cervical cancer. Population-based screening programs have improved detection of the preinvasive and early stages of cancer, what have led up to decline in the incidence of advanced disease. Nonetheless, the verification of the presented results on the basis of a greater material is by all means justified. Undoubtedly, the advantage of this contribution is its prospective form and the homogeneity of the material. In comparison with the repository databases, the presented results are derived from the single institution, which represent coherent therapeutic concept for the cervical cancer treatment. An experienced team of gynecological surgeons, applying the rules of established operating school, eliminates the risk of randomness of the results and enhances their reliability.
